# Calculating the Social Return on Investment of a Japanese Professional Soccer Team's Corporate Social Responsibility Activities

**DOI:** 10.3389/fspor.2021.736595

**Published:** 2022-01-17

**Authors:** Daichi Oshimi, Shiro Yamaguchi, Takayuki Fukuhara, Yoshifumi Tagami

**Affiliations:** ^1^School of Physical Education, Tokai University, Hiratsuka, Japan; ^2^Faculty of Human and Social Sciences, University of Marketing and Distribution Sciences, Kobe, Japan; ^3^Department of Movement and Sport Sciences, Vrije Universiteit Brussel, Brussel, Belgium; ^4^Art and Sports Business Course, Hokkaido University of Education, Sapporo, Japan; ^5^SDM Research Institute, Graduate School of System Design and Management, Keio University, Yokohama, Japan

**Keywords:** social return on investment (SROI), corporate social responsibility (CSR), social impact, community soccer/physical activity program, non-market benefits, Japanese professional soccer team

## Abstract

In response to the lack of evidence for visualizing the monetary value of professional sport teams' CSR-related social impact, this study aimed to calculate the social impact of a Japanese professional soccer team's corporate social responsibility (CSR) activity (i.e., community soccer/physical activity program) by using a social return on investment (SROI) framework. Specifically, Matsumoto Yamaga Football Club was used for the estimation. This professional soccer team was ranked in the top division of the league (J1) during the estimation period and engaged in CSR activities at 129 community soccer schools in a year (2019–2020). The SROI calculation involved five stages: (1) identifying key stakeholders, (2) mapping inputs, outputs, and outcomes, (3) measuring and valuing outcomes, (4) establishing impact, and (5) calculating SROI. For the first step, we specified seven major stakeholders (soccer team, nursery school children, parents, coaches, teachers, local governments, and local football associations) and conducted interview investigations with each stakeholder. Our result showed that the social value of the teams' targeted community soccer school was USD 54,160, and the total financial and non-financial inputs to the school were USD 10,134, meaning an SROI ratio of 5.3. This means that for every USD invested in a community soccer school, social benefit worth USD 5.3 was generated. This study contributes to advancing social impact research in sport by shedding light on the monetary value of the social impact of professional sport teams' CSR activities. In addition, it also has practical implications for team managers looking to utilize CSR activities as a management strategy, through cost-effective investment and optimization of resources.

## Introduction

Since the 1960s, there has existed a long history that can be used to estimate the social impacts of sport and leisure activities such as improved personal, physical, and mental health or community benefits (e.g., social cohesion; Davies et al., 2020). Although several scholars have clarified these effects using Likert scales or rankings (Davies et al., 2019), these studies have been criticized for their ambiguity (in-tangibility) due to biased responses from subjects (Fujiwara, 2014). Corporate social responsibility (CSR) includes economic, legal, ethical, and philanthropic components (Carroll, 1991), and has emerged as a business driver in the last decades (Fifka and Jaeger, 2020). Recent literature suggests that measuring, managing, and communicating about corporate social and environmental activities are necessary for competitive corporate sustainability performances (Grewal and Serafeim, 2020). However, Grewal and Serafeim also pointed out that measuring the impacts is the least developed area among them, because accounting experts have spent little effort to finding how to measure the performances. This difficulty in quantifying the outcomes of the activities has perhaps been due to the intangible nature of their impacts (Lombardo et al., 2019). Furthermore, past researches tend to focus on *inputs (i.e., efforts or investments)* rather than *outcomes* even though outcome-based metrics are more strongly correlated with future productivity and growth (Kotsantonis and Serafeim, 2020).

Recently, some studies have clarified the positive relationship between CSR practices and financial performance, and more companies are trying to implement CSR practices in their business strategy (e.g., Hategan et al., 2018; Simionescu and Dumitrescu, 2018; Moyo et al., 2021). However, CSR in the sport management literature is still immature, and further research is required to develop CSR evaluation models for effective decision-making within organizations (Kihl et al., 2014a; Walzel et al., 2018; Carlini et al., 2021). In this stream, social return on investment (SROI) has received growing attention as a method of calculating the monetary value of an activity or project's social impact. Monetizing impacts enable business managers and investors to understand, compare, and analyze the impacts for better decision-making (Kotsantonis and Serafeim, 2020). SROI is regarded one of the most established social impact assessment methods (Mulgan, 2010; Lombardo et al., 2019) and a framework to measure the net social outcomes of an activity or organization (Nicholls et al., 2012; Harlock, 2013). Despite this, to the best of our knowledge, only a few empirical papers have conducted SROI studies in the sport management literature (Davies et al., 2019, 2020; Lombardo et al., 2019). Although SROI analyses of CSR activities by professional sport teams have been conducted (Lombardo et al., 2019), their comprehensive analyses captured not only the teams' CSR activities, but also tourist revenue from their professional football games. Furthermore, their philanthropic activities are described ambiguously; it is not clear which CSR activities cultivate social benefits. To understand the monetary value of CSR activities through sport further, more empirical research targeting one specific CSR activity is necessary.

Thus, the purpose of this study is to calculate the social impact of a single CSR activity (i.e., community soccer/physical activity program) by a professional Japanese soccer team using an SROI framework. By virtue of its focus and methodology, the current study, therefore, contributes to progressing social impact research by shedding light on the monetary value of a single CSR activity for professional sport teams. This enables us to understand the monetary value of a social activity in a clear way by targeting a single CSR program. Furthermore, the SROI framework sheds light on the “outcomes” of a social activity, which are more correlated with future productivity than inputs, contributing to the visualization of the social impacts of CSR activity. This could be one of the solutions to the ambiguity (in-tangibility) of social impacts, and we expect to show another method of measuring estimate social activities.

## Literature Review

### Socio-Economic Impacts of Sport

Taylor et al. (2015) summarized the impacts of sport into five areas: health, crime, education, social capital, and subjective well-being, and concluded that the most robust evidence is accumulated in health benefits (i.e., physical and mental health). While evidence of the impact of social capital on education (e.g., attainment) and crime (prosocial behavior) exists, social capital is less convincing than other benefits. In sport-science literature, the number of studies focusing on subjective well-being has been limited; yet this topic is one of the hot issues in the sport/event and tourism management literature (e.g., Sato et al., 2020; Vada et al., 2020; Wicker and Downward, 2020; Wendtlandt and Wicker, 2021). There are also studies estimating the socio-economic benefits of sport teams and stadiums/arenas in the community. For instance, Agha and Coates (2015) found that rents rose from 6 to 8% because of minor league baseball teams in the U.S. In addition, several studies have investigated the willingness-to-pay for the area/stadium and teams in exchange for receiving intangible benefits, such as civic pride and excitement (e.g., Johnson and Whitehead, 2000; Johnson et al., 2001; Santo, 2007). Baumann et al. (2012) and Pyun (2019) examined the impacts of sporting events/franchises on crime rates, and Pyun (2019) found a 7% increase per month in the number of violent crimes in the city. While these studies have contributed to visualizing the socio-economic impacts of sport on the community or individual, there are some methodological issues, especially related to the ambiguity (in-tangibility) of social impacts due to biased responses from subjects (Fujiwara, 2014).

### CSR in Professional Sport

While CSR has increased practical and academic attention toward creating value between business, firms, and society (e.g., Kolyperas et al., 2016; Hills et al., 2019), CSR research in professional sport is a relatively new study area (Breitbarth et al., 2011). CSR includes all aspects of an organization's activities that contribute to creating social and environmental benefits (Kulczycki and Koenigstorfer, 2016). The recent competitive business environment must consider social involvement through CSR (e.g., Anagnostopoulos et al., 2014; Kihl et al., 2014a; Moyo et al., 2021), as this could provide a competitive advantage against competitors (Porter and Kramer, 2006). Specifically, measuring, managing, and communicating about corporate social and environmental activities is necessary for competitive corporate sustainability performances, and measuring the impacts is the least developed area among them (Grewal and Serafeim, 2020). Past studies have investigated CSR from several perspectives, such as the relationship between CSR activities and the development of fan–team relationships (e.g., Walker and Kent, 2009; Kim et al., 2015; Lacey and Kennett-Hensel, 2016; Liu et al., 2019; Chen and Lin, 2021), environmental sustainability development (e.g., Inoue and Kent, 2012; Trendafilova et al., 2013), program benefits or social impacts on stakeholders (e.g., Kihl et al., 2014b; Walker et al., 2017; Riggin et al., 2019) and the determinants, pressures, or motives of CSR (e.g., Babiak and Wolfe, 2009; Babiak and Trendafilova, 2011). Regarding the benefits of CSR in professional football, the review literature has summarized the following nine outcomes of CSR: brand image, reputation, identification, new partners, new supporters, financial value, cultural value, human value, and reassurance (Fifka and Jaeger, 2020). However, the financial evidence of CSR activities by professional sport has been limited (Walzel et al., 2018). Some literature has investigated the relationship between CSR and financial performance (e.g., Inoue et al., 2011; Hategan et al., 2018; Simionescu and Dumitrescu, 2018), while Breitbarth et al. (2011) has conceptualized the CSR Performance Scorecard, which is useful for increasing operational transparency and foster stakeholder communications. Nevertheless, little empirical evidence has been developed with regard to the financial evaluation of CSR activities by professional sport. Monetizing impacts enable business managers and investors to make better decisions based on said impacts (Kotsantonis and Serafeim, 2020).

### SROI in Sport

In these streams, Lombardo et al. (2019) calculated the SROI of a professional soccer team, becoming the first study to evaluate the benefits of football clubs and their philanthropic organizations to society. They identified eight stakeholders related to their activities and quantified the resultant socio-economic benefits—such as the enhanced attractiveness of their city, stakeholders' improved psychological conditions and skills, and revenue increase—into monetary value. They utilized the life-effectiveness questionnaire to estimate the stakeholders' psychological condition and concluded that the social impact created during the championship (4 months) amounts to approximately EUR 44 million against a financial investment of EUR 15 million, producing an SROI ratio of 2.98:1. This means that for every euro invested by the football club, about EUR 3 of social value is created.

Davies et al. (2019) estimated the SROI of physical activity in England at the national level by identifying several stakeholders such as public sector institutions (e.g., central/local governments, schools, public organizations) and private institutions (e.g., companies), consumers (e.g., participants, volunteers), and philanthropic organizations. In addition to interview investigations, they utilized secondary data (e.g., from Active People Surveys and Taking Part Surveys) and previous literature to calculate the monetary value of sport participation's social value. As a result, Davies et al. (2019) found that the social value of sport participation in England in 2013/14 was GBP 44.8 billion, and the total financial and non-financial inputs to sport were GBP 23.5 billion, giving an SROI ratio of 1.91. In addition, Davies et al. (2020) estimated the SROI of a specific physical activity program (Physical Activity Referral Scheme) in 12 community sport and leisure facilities in Sheffield. They calculated GBP 21.67 million and GBP 0.26 million, respectively, and found that for every GBP 1 spent, a SROI of between GBP 1.20 and GBP 3.42 in community sport and leisure facilities was gained. Social value includes socio-health-related outcomes, such as those related to health (e.g., cancer, dementia), crime, education (e.g., attainment and human capital enhancement), subjective well-being, and human resources.

Although these novel approaches have the potential to shed light on the monetary value of social impact on communities, SROI analysis targeting sport teams' CSR activities is still very limited.

## Theoretical Background of SROI

There are several social impact assessment tools, including social accounting and auditing, resource analysis, and social return assessment. One of the characteristics of SROI is that it mainly focuses on the social outcomes (e.g., well-being) of social activities for which no market values exist and includes several stakeholders' perspectives (Keane et al., 2019). The theoretical background of SROI is based on the theory of change, which is a methodology used to describe the logical sequence of an initiative from inputs to outcomes (Vogel, 2012). The continuum of elements is represented starting from inputs and outputs through to outcomes and they collectively describe how and why a desired change is expected to occur in a certain context (Lombardo et al., 2019). SROI is based on economic evaluation frameworks, particularly the cost-benefit analysis (CBA). While CBA focuses only on economic cost and benefits, SROI includes more comprehensive perspectives to evaluate various social impacts (Pathak and Dattani, 2014; Banke-Thomas et al., 2015) by considering the extensive use of stakeholders related to the activity (King, 2014).

The advantages of SROI for management (e.g., policy makers and project managers) are that it (1) strengthens accountability and communication with several stakeholders (Mook et al., 2015), (2) improves resource optimization through calculating cost-effectiveness (Maier et al., 2015; Watson et al., 2016), (3) enhances community awareness of an organization's profile (King, 2014), and (4) develops organizational (or program) sustainability (Nicholls et al., 2012). Although SROI also has several disadvantages, such as the lack of guidance on estimating long-term impacts (Fujiwara, 2014), potential bias for over- or under-claiming, or the tendency to accentuate the positive benefits (King, 2014), these issues are not unique to SROI such as CBA (Davies et al., 2019). Ensuring transparency when calculating SROI could be a solution to safeguarding the validity of the estimation.

## Method

### Research Context

A case study approach was applied to the current research, which is appropriate for an in-depth investigation to explore complex social phenomena (Yin, 2009). Although the case study approach is unlikely to allow for theoretical and statistical generalizations (Yin, 2009), the results are well-suited for management inquiry (Larsson, 1993). The case study requires strict research design, which consists of research problems and propositions, units of analysis, logic that connects the data to propositions, and criteria for interpreting survey results (Yin, 2009). Therefore, it is suitable to use the SROI framework, which contains these assumptions, to visualize the social impact of a Japanese professional soccer team's CSR activity.

The Japan Professional Football League (henceforth J. League) was founded in 1991 and began its first season with 10 clubs in 1993 (J. League, n.d.). In addition to the top division of the league (i.e., J1), they established Division 2 (i.e., J2), dividing the league into 18 (J1) and 10 (J2) clubs in 1999. Five years later, Division 3 was launched, and as a result, by 2014, the J. League had 51 clubs (18 [J1], 22 [J2], and 11 clubs [J3]). At present, the number of clubs is 55, and the J. League aims to create a society in which everyone can enjoy not only soccer, but also any sport. To achieve this goal, the J. League set up the overarching 100-year vision to become an agent of change by fostering a sport culture in Japan rooted in community-based sport clubs. Thus, each club needs to create an environment for a healthy, fully integrated sport culture for children based on their vision.

For instance, each club has been providing hometown activities to their host city, and consequently made great progress in fostering the development of Japan's sporting culture to improve the mentally and physically health of the people. Recently, the J. League launched another social corporation program called “SHAREN” to foster partnerships with public/private organizations for community development in each hometown since 2018 (J League, 2018). In order to enhance the partnership, it is necessary for them to visualize the program's impacts/benefits on the community including in-visible (social) benefits. Thus, the J. League's hometown activity is the right case to apply the SROI framework to visualize (monetize) its impacts. In our study, the Matsumoto Yamaga Football Club (MYFC) was used for the estimation. Specifically, we targeted their CSR activities, which are centered on their community soccer and physical activity programs in their local city (hometown). The program entails the MYFC coaches delivering a free 30-minute enjoyable soccer or physical activity lesson to local children in their schools. The program was partially commissioned by the local football association for the development of sport/soccer in their region, and they provided 129 community soccer/physical activity programs in the 2019 season. MYFC was established in 2004 based on Yamaga Soccer Club, which was founded in 1965 (MYFC, n.d.). In 2014, the club finished second in Division 2 and was promoted to Division 1 for the first time. It was during this time (from 2019 to 2020) of being in Division 1 that this study was carried out.^1^ Their hometown activities spread across Nagano prefecture, including nine specific hometowns such as Matsumoto City, Shiojiri City, Yamagata Village, Azumino City, Omachi City, Ikeda City, Ikusaka Village, Minowa Town, and Asahi Village. The reason our study selected the MYFC was that the club secured the top score in management soundness within their league (Deloitte, 2019) and provided a sufficient number of hometown activities (649 times in a year) among the other J. League teams. Thus, MYFC is an appropriate subject for investigation.

### Data Procedure

**Figure 1:**
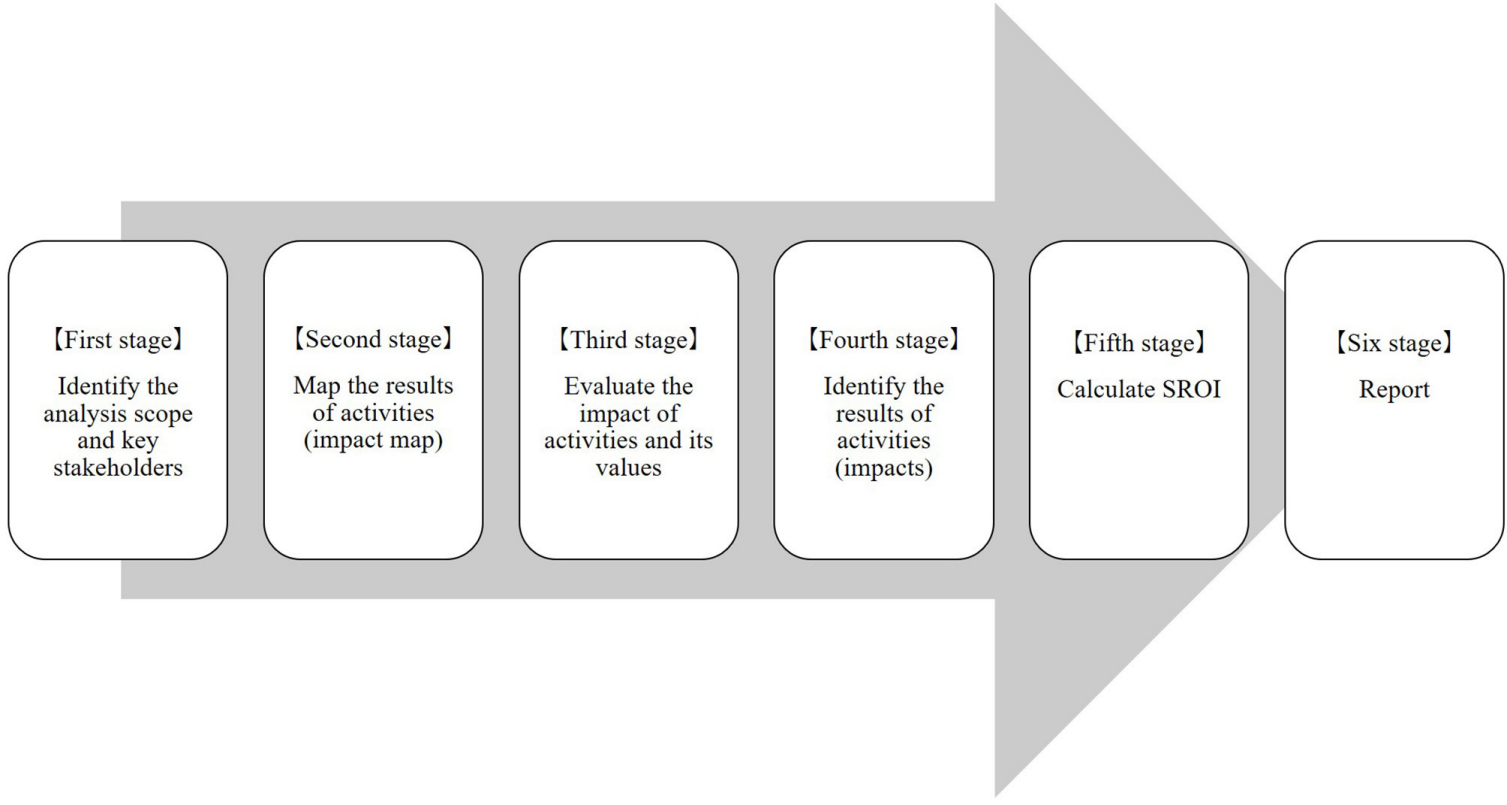
The stages of SROI.

The calculation of SROI is based on previous literature (Nicholls et al., 2012; Davies et al., 2019) and was carried out in six stages. These are: (1) identify the scope of the analysis and key stakeholders, (2) map the results of activities (impact map), (3) evaluate the impact of activities and their values, (4) identify the results of activities (impacts), (5) calculate SROI, and (6) report (Figure 1). The SROI analysis was conducted over a period of ~9 months (April 2020 to January 2021).

According to Lombardo et al. (2019), the SROI methodology describes how change is being created, placing monetary value on the change. As explained above, this way of thinking is reflected in the theory of change (Vogel, 2012). In our study, the SROI was calculated based on the following formula:


$$SROI\;=\;\frac{Quantification\;of\;outcome\;(incudes\;financial\;proxy)}{Quantification\;of\;input}.$$

Furthermore, sensitivity analysis was conducted based on the variation of different assumptions, which enables us to assess the sensitivity of the SROI to the initial hypothesis, depending on the different scenarios.

The assumption for calculating the SROI of the community soccer/physical activity program is based on the fact that physical activity programs have positive impacts on young children, such as on their cognitive function (Sibley and Etnier, 2003). However, such evidence is gathered from other countries, whereas little evidence has been accumulated in Japan. Specifically, little evidence was found on the CSR activities (e.g., community soccer school) of professional sport teams. Thus, this could be a reasonable assumption for estimating the SROI of the program. Moreover, these programs are common to other Japanese professional soccer teams as a CSR activities (J League, 2020). Regarding their advantages, the programs are accessible to the participants because most of them are held in the participants' own schools and neighborhoods. Given that accessibility to the program is important for children to perform physical activity (Sallis et al., 2000), the program's application in the SROI analysis is justified to consider its feasibility in other similar contexts. Finally, since the program was partially commissioned by the local football associations' annual budget, it is reasonable to limit the estimation period to a single year for examining the program's accountability to the stakeholder.

## Results

In this section, the results of the SROI analysis are presented based on the five stages described in the previous section^2^.

### Stage 1: Identify the Analysis Scope and Key Stakeholders

In the first stage, the authors conducted a discussion to determine the scope of the analysis and identify key stakeholders. With regard to the scope of the analysis, the following items were considered; the purpose of the analysis, target activity, content (whether it is worth it to analyze or not?), key stakeholders, and analysis period.

**Figure 2:**
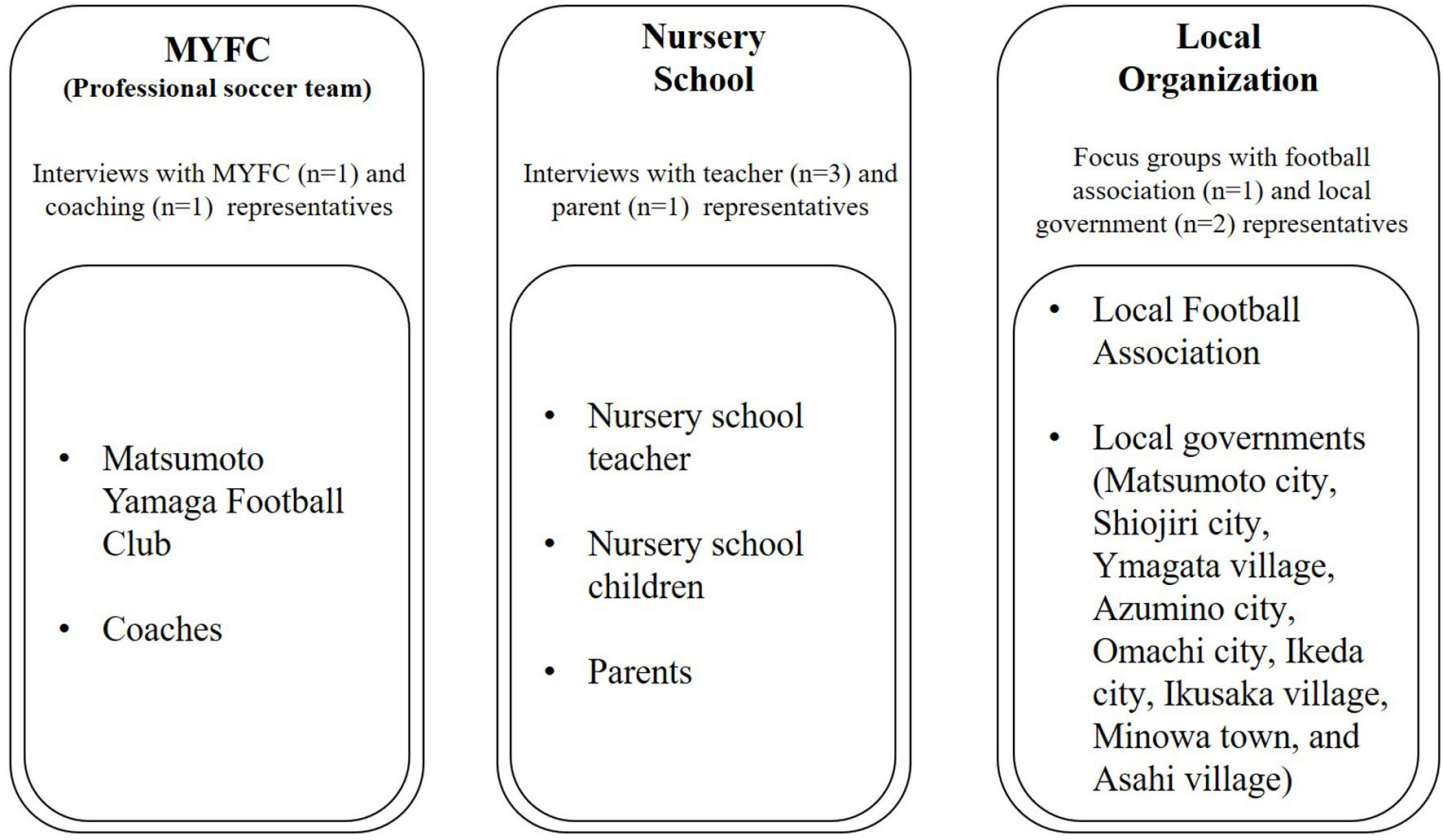
Stakeholder map.

Next, we identified stakeholders that are engaged in the program based on the definition by Davies et al. (2019), which identifies stakeholders as people or organizations that influence or experience change because of the program. As a result of the desk review with the chief staff in charge of the program, three stakeholder groups were identified, as shown in Figure 2.

In the first group, MYFC and coaches were extracted as internal stakeholders. They are running the community soccer/physical activity programs, and they could obtain some social benefits from their activities. In the second group, nursery school teachers, children, and the children's parents were extracted as external stakeholders. This program was carried out for nursery school children and they were the main targeted participants of the activities. As a final group, local football associations and local governments (Matsumoto City, Shiojiri City, Yamagata Village, Azumino City, Omachi City, Ikeda City, Ikusaka Village, Minowa Town, and Asahi Village) were retrieved as external stakeholders. The local football associations provided MYFC with funds for the program as compensation for the promotional sport and soccer activities in their region. MYFC delivers the program in each region as a return on funding. Local governments also play a role in sport development for the health of residents, including kids; thus, they are also regarded as key stakeholders in the program. As a result, we specified seven key stakeholders (i.e., MYFC, coaches, nursery school teacher, nursery school children, parents, local football associations, and local governments) from the three groups to proceed with further analysis.

### Stage 2: Map the Results of Activities

In the second stage, we created the impact map of the activities by performing eight semi-structured interviews (including two focus group interviews) in total with nine stakeholders on October 6, 13, and 27, 2020 (see Figure 2). Mapping the outcome of activities is of great importance for SROI analysis (Davies et al., 2019) and the impact map (i.e., the input, output, and outcome) was visualized by following the logic model. Each interview lasted 40 to 90 mins and was digitally recorded. The following areas were explored *via* interview questions: (1) relationship with the MYFC's community soccer/physical activity program and (2) outcome (change) by the program. We also added other questions depending on the stakeholders' answers and collected supplemental materials from websites, archives, and other references.

**Figure 3:**
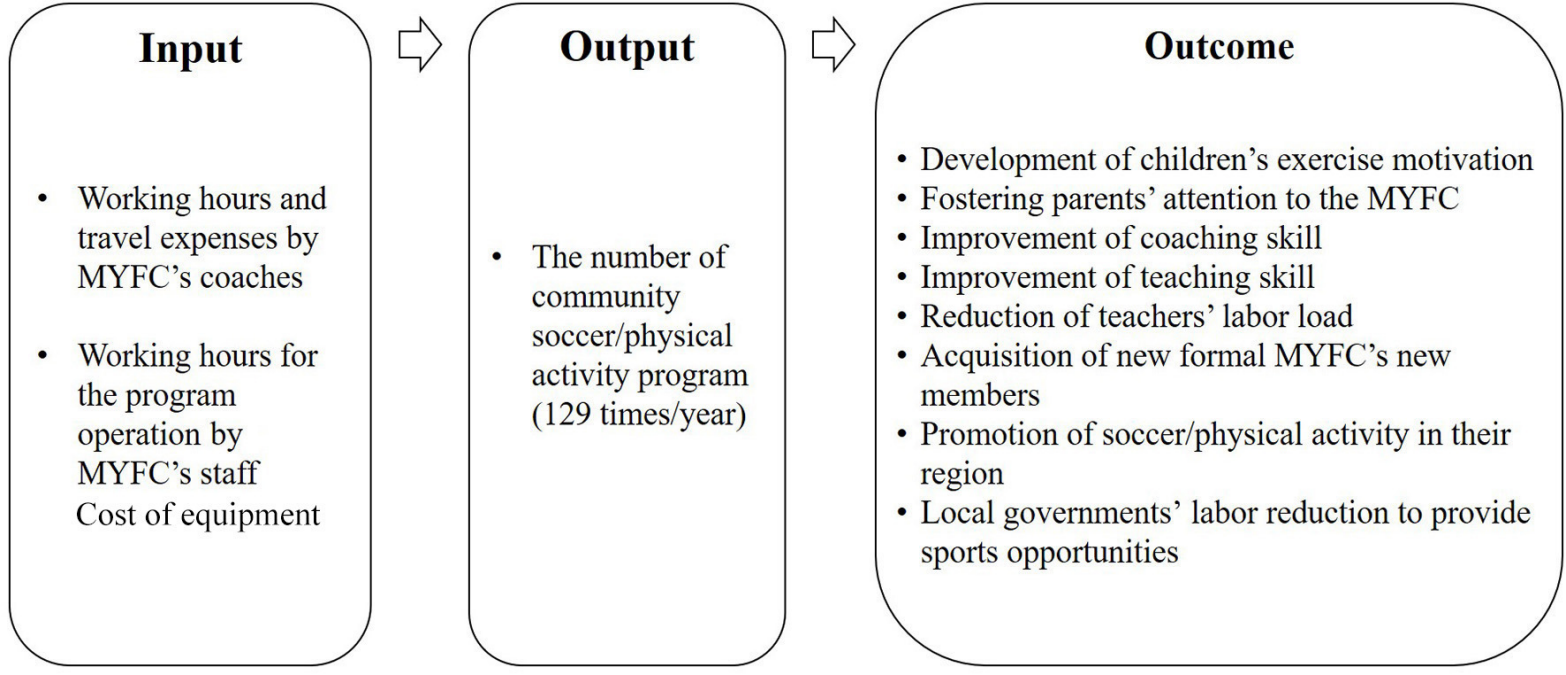
Overview of the impact map.

The impact map was created based on the interviews. As an input, cost for coaches' working hours (USD 48.54 ×129 times = USD 6,262.136), travel expenses (USD 9.71 × 129 times = USD 1252.427), cost of equipment (USD 79.551), and staff working hours for the program operation (USD 19.69 × 129 times = USD 2,539.781) were included in total USD 10,133.895.^3^ With regard to output, the number of community soccer/physical activity programs (129 times/year) was included. As an outcome, eight impacts were extracted: (1) development of children's exercise motivation; (2) fostering parents' attention to the MYFC; (3) improvement of coaching skills; (4) improvement of teaching skills; (5) reduction of teachers' labor load; (6) acquisition of new formal MYFC club members; (7) promotion of soccer/physical activity in their region; and (8) local governments' labor reduction to provide sport opportunities. Figure 3 shows an overview of the impact map used in this study.

### Stages 3 and 4: Evaluate the Impact of Activities and Its Values and Identify the Results of Activities (Impacts)

In the third and fourth stages, we confirmed and identified the impact of actual activities and their values. To quantify the eight outcomes, the authors discussed the best financial proxy to describe the outcomes (see Table 1).

**Table 1:** Results of social return on investment analysis.

**Stakeholder**	**Outcomes**	**Financial proxy**	**Unit change amount (USD 1 = JPY 103)**	**Outcome amount**	**Impact amount (USD 1 = JPY 103)**
Nursery school children	**Development of exercise motivation** Learn how to move their body Stop being afraid of the ball/Come to like exercise	Average price for children's private sport program in Matsumoto city (Per half-hours)	10.864	3,450 children	37,480.800
Parents	**Fostering attention to the Matsumoto Yamaga Football Club (MYFC)** View their children's photo on the website of MYFC	Price for internet banner ad of MYFC (Per click)	0.002	3,450 parents	6.203
Coaches	**Improvement of coaching skill** Learn coaching methods for kids and their application to adults	Tuition for the lecture of kids' coaching license in the local football association (Per half-hours)	6.476	129 times ×2 coaches	1,670.738
Nursery school teachers	**Improvement of teaching skill** Learn teaching methods such as how to attract children's attention	Tuition for the lecture of kids' coaching license in the local football association (Per half-hours)	6.476	129 times ×2 teachers	1,670.738
	**Reduction of labor load** Adopt the program menu to their daily physical activity programs	Salaries for public servants in Matsumoto city (Per hour)	19.688	129 times ×1.5 h	3,809.672
MYFC	**Acquisition of formal MYFC's new members** Acquisition of new members through the program	Admission and annual membership fee (Per person)	466.019	10 members	4,660.194
Local football associations	**Promotion of soccer/physical activity in their region** Business trust agreement with the MYFC for the further development of sports/soccer in their region	Expenses for commission to MYFC	2,427.184	–	2,427.184
Local governments	**Labor reduction to provide sports opportunities** Working hours reduction of local government employee for sport development in their region	Salaries for public servants in each city ^a^ (Ikeda, Matsumoto, Minowa, Azumino, Shiojiri, Omachi, Higashichikuma, Tatsuno, and Anan) (Per half-hours)	Ikeda: 9.035 Matsumoto: 9.844 Minowa: 9.023 Azumino: 9.400 Shiojiri: 8.723 Omachi: 9.035 Higashichikuma: 9.381 Tatsuno: 8.795 Anan: 8.437	129 times (Ikeda: 2, Matsumoto: 61, Minowa: 24, Azumino: 17, Shiojiri: 11, Omachi: 6, Higashichikuma: 4, Tatsuno: 3, and Anan: 1) ×2persons ×30min	2,434.835
**Total value of all outcomes**					**54,160.364**

*a = The unit change for local governments amount differs depending on each municipality*.

*Development of children's exercise motivation* was calculated by the average price (unit change amount: per half-hour^4^) of children's participation in a private sport program in Matsumoto City (USD 10.86 ×3,450 children = USD 37,480.800). Through an interview investigation with the nursery school teacher and parents, we found that children enjoyed the physical activity program more than daily programs performed by the nursery school teachers because of the coaches' prominent teaching skills. The following representative responses were obtained from the teachers and parents:

*They have good skills in teaching the children how to move their bodies and handle the ball. He loved the program because he had never experienced such a fun program in the nursery school*.*Their coaching method was great*. *My child had so much fun and never got bored. Thanks to their coaching, he is getting used to playing/kicking a ball and is getting over his fear of [playing with] the ball*.

Thus, as a financial proxy, the program could provide a basis for considering the price of private sport programs for children. *Fostering parents' attention to MYFC* was substituted for the price of the Internet banner advertisement on the MYFC official homepage (USD 0.002 × 3,450 parents = USD 6.203). We found that parents (at least one of each child's parents) looked at their children's pictures in the program on the MYFC official website, which could increase their attention to the club. The one-click banner advertisement on their website was worth USD 0.002; thus, we exchanged the attention (click on the website) increase into the advertisement value.

*Improvement of coaching skills* was replaced with the tuition fees for coaches to undergo child training lectures (unit change amount: per half-hour) in the local football association (USD 6.476 × 129 times × 2 persons = USD 1,670.738). Through the program sessions, coaches have opportunities to develop their coaching skills, and they mentioned that these skills are applicable not only for children, but also for adults. Here, the financial proxy is validated by the price of the Kids Leader Training Course in a local city.

*Improvement of teaching skills of nursery school teachers* was also converted to the tuition fees for teachers to undergo child training lectures (unit change amount: per half-hour) in local football associations (USD 6.476 × 129 times × 2 teachers = USD 1,670.738). In addition, *the reduction of labor load* for them was calculated as a proxy by considering public servants' salaries (unit change amount: per hour) in Matsumoto (USD 19.688 × 129 times × 1.5 h^5^ = USD 3,809.672). During the interview, the following representative response was obtained from a nursery school teacher:

*We learned from their [coaches'] training and adopted the program in the nursery physical activity program because the program is very useful as a children's physical activity menu and can attract children's attention*.

The teachers learned coaching skills by observing and helping the program. Moreover, we found that they utilize the program's contents in their daily physical activity programs in class or other events owing to their usefulness. This leads to a reduction in their labor load, as they need not prepare anew for their physical activity programs.

*Acquisition of formal MYFC's new club members* was substituted for admission and the annual membership fee of MYFC (USD 466.019 × 10 members = USD 4,660.194). Through the interview investigation, we found that they could acquire 10 new members of the formal/regular-basis MYFC soccer school because of the program. The following representative response was obtained from one of the parents:

*My child participated in the program and joined the formal MYFC soccer school afterward. He naturally loves exercising, and compared to other soccer schools, this school provides a unique program for children*.

*Promotion of soccer/physical activity in their region* was exchanged for the funds provided to the MYFC (USD 2,427.184) by local football associations. Local football associations have signed a business trust agreement with the MYFC to spend USD 2,427.184 funding them. Their aims are to further develop sport and soccer in these regions. In return for the fund, the MYFC delivers the sport development program on behalf of the local football associations. Finally, *local governments' labor reduction to provide sport opportunities* (unit change amount: per half-hour) was accounted for by public servants (USD 2,434.835)^6^. Through these activities, local governments could save their working hours by having the MYFC provide sport opportunities to residents in their stead. As a local government, they have a responsibility to provide sport opportunities to promote residents' health; thus, the MYFC program contributes to reducing local government working hours in each of the nine hometown cities. Table 1 summarizes the results of the SROI analysis. No negative impact was found during the interviews.

### Stage 5: Calculate SROI

The SROI was calculated in the fifth stage based on the estimation process (total value of all outcomes/total inputs). As a result, the total social value of the community soccer school was USD 54,160 (see Table 1), which is the sum of eight outcomes, listed as follows: children's development of exercise motivation (USD 37,480), fostering parents' attention to the MYFC (USD 6), improvement of coaching skill (USD 1,671), improvement of teaching skill (USD 1,671), reduction of teachers' labor load (USD 3,810), acquisition of formal MYFC's new members (USD 4,660), promotion of soccer/physical activity by local football associations (USD 2,427), and local governments' labor reduction (USD 2,435). On the other hand, the total financial and non- financial inputs to the school were USD 10,134 calculated in Stage 2, which was the sum of four inputs, listed as follows: coaches' working hours (USD 6,262), coaches' travel expenses (USD 1,252), cost of equipment (USD 80), and staffs' working hour for the program operation (USD 2,540). Therefore, based on these totals, an SROI ratio of 5.3 was calculated, which means that for every USD invested in a community soccer school, USD 5.3 worth of social benefit was generated. Since the estimation period is just 1 year, adjusting for the duration of an outcome and drop off^7^ was not required. Furthermore, considering that there are no similar soccer/physical activity programs in the region and several confounding factors are inherently embedded in sports and exercise (Davies et al., 2019), deadweight^8^ and attribution were not considered in the analysis. Displacement^9^ was also not considered because of the uniqueness of the program in the community and the uncertainty of the substitution effects such as between leisure activities and sports (Davies et al., 2019).

#### Stage 5-1: Sensitivity Analysis

A sensitivity analysis was performed based on the previous literature (Lombardo et al., 2019) by arranging variations of different possible assumptions in our research context (Table 2). The analysis enables us to assess the sensitivity of the SROI to the initial hypothesis, depending on the different scenarios. The analysis was conducted by considering the changes in MYFC coaches' cost of working hours and the number of programs, which are the main inputs in this program and the only relevant variables for MYFC. We assumed that the variation ranges from−20% to +20%. In each case, the outcome shows that the program produces positive social value. The most prudential case is 4.64 (assuming 120% of the coaches' cost for work hours and 120% of the number of programs), while the best scenario is 6.29 (assuming 80% of the coaches' cost for work hours and 80% of the number of programs).

**Table 2:** Results of sensitivity analysis.

**Coaches' cost for work hours (%)**	**Number of programs (%)**	**SROI ratio**
100	100	5.34
80	80	6.29
80	120	4.90
120	80	5.95
120	120	4.64

*SROI, social return on investment*.

## Discussion

The purpose of this study was to calculate the social impact of a professional Japanese soccer team's CSR activity (i.e., community soccer/physical activity program) using an SROI framework. The study contributes to advancing social impact research in sport by shedding light on the monetary value of the social impact of specific CSR activities by professional sport teams. This novel approach could be one of the solutions to the problem of ambiguity of social impact that has plagued past studies in this field (Fujiwara, 2014), in particular the lack of evidences in “measuring outcomes (not input)” of the social activities (Grewal and Serafeim, 2020; Kotsantonis and Serafeim, 2020). In addition, our research adds to the evidence of economic/financial responsibility in CSR activities by professional sport teams, which has been limited to CSR research by professional sport organizations (Walzel et al., 2018). In particular, the current study targeted one specific activity (i.e., community soccer/physical activity program), which enabled us to identify the socio-economic value of CSR activities by professional sport. Considering the growing attention toward the strategic relationship between business and society (e.g., Anagnostopoulos et al., 2014; Kihl et al., 2014a; Kolyperas et al., 2016; Hills et al., 2019; Moyo et al., 2021), estimating the monetary value of CSR activity might be a trigger to fostering a stronger mutual relationship between professional sport teams and their stakeholders (e.g., staff, sponsor, residents). Moreover, the financial value might produce a synergistic effect with other CSR outcomes such as brand image/reputation development or obtaining new partners/supporters (Fifka and Jaeger, 2020). This develops organizational sustainability and could provide a competitive advantage against competitors (Porter and Kramer, 2006).

The results have several implications for professional sport teams and leagues. First, the SROI framework could be applied to sport teams' CSR activity (i.e., community soccer/physical activity program), which indicates that these activities could create not only social, but also monetary value in the community. For example, cost savings for nursery teachers and staff of local community/government institutions are important considering the limited resources of nurseries school and communities. In particular, human resources in the local government are limited, and soccer schools are a valuable opportunity for providing children in the community with enjoyable sport activities. Thus, it might be useful to promote the program not only for children but also for teachers/schools and local sport organization/government by visualizing the cost saving benefits of the program. Second, SROI could help explain teams' accountability to their stakeholders (e.g., sponsors and local federations), which contributes to enhancing the teams' community awareness (King, 2014). For instance, the survey visualized the monetary value of the enjoyment of physical activities, which could appeal to potential sponsoring companies (e.g., the health/education industry). In addition, the promotion of soccer and physical activities contributes to attracting attention from local soccer federations and plays an important role in promoting soccer and physical activities in teams' local communities. Therefore, these socio-economic benefits should be highlighted to various stakeholders (e.g., public/private or sport organization) in their community to enhance their community awareness. This could create reasons to obtain more tangible and intangible support (e.g., fund, subsidy, human resources) from several organizations. Third, the results provide evidence that team managers use CSR activities as a management strategy, which enables managers to make these activities cost-effective by optimizing their resources. For instance, the sensitivity analysis found that increasing the number of programs lowers the SROI ratio within a ± 20% range (Table 2), and the team's managers could use the results to develop their programs further. For example, given the coach's higher cost of working hours, simply increasing the number of programs does not mean higher SROI ratios. Therefore, team managers need to consider the cost-effectiveness of the program. Instead, developing the quality (not quantity) of the program might be a better option for delivering their programs more effectively. Moreover, the results of the current study indicated that the impacts of the program on nursery school children and teachers were greater than those on other stakeholders, and club managers could utilize this output to consider their strategy of CSR activities by managing their resources. Specifically, they could use the results to estimate whether the activities efficiently/positively affect the main targets in the program. If the activities are not able to effectively deliver benefits to their main stakeholder, club managers might need to reconsider the program contents/operation to optimize its impact on each stakeholder.

## Conclusion

The current study is an advanced research paper that has applied the SROI framework to the sport industry. Even though the framework is one of the most established social impact assessment methods (Mulgan, 2010; Lombardo et al., 2019), the literature shows that only a few SROI based studies have been conducted (Davies et al., 2019, 2020; Lombardo et al., 2019). Specifically, studies focusing on one specific CSR activity are sparse. Thus, the current study extends the literature on the socio-economic impact of sport in the context of CSR activities by professional sports. In particular, the current case analysis considering multiple stakeholders enables us to consider management inquiry of the CSR activities of a professional sport team. However, it still suffers from some limitations. The primary limitation of the current study is the issue of generalization. As the current study is focused on a specific soccer/physical activity program, it cannot automatically be generalized to all other soccer/physical activity programs. Moreover, several indicators (e.g., stakeholder, outcomes, financial proxy), which are necessary in the SROI framework might differ depending on the context or programs. Thus, replicating/accumulating more cases is necessary to find common/reliable indicators for future SROI based research. Additionally, adjusting each indicator based on price fluctuations might be required in a longitudinal research design. Furthermore, we could not use a pre-post research design or set a controlled group, which could have enabled us to obtain more robust results; these approaches should be considered in future studies. Moreover, as the main recipients of the program are nursery school children, we could not include socio-psychological benefits (e.g., educational benefits and well-being) because of the difficulty of handling them as research objects. Lastly, although no negative impacts (e.g., injuries) were found from the interview results, some potential negative outcomes may have been excluded from the estimation process. This may be due to the lack of examination and empirical evidence on the effect of the program on the participants (i.e., nursery school children). Further research using the SROI approach is necessary to obtain more valid and common financial proxies. For instance, applying a questionnaire survey to estimate the psychological effects of the program could be a useful way to visualize them. This approach enabled us to capture other effects and shed light on the comprehensive monetary value of the social impact of CSR activities by sport teams. In addition, examining the relationship between SROI and financial performance could be an area for future research in SROI intended to make it a common financial indicator.

## Data Availability Statement

The raw data supporting the conclusions of this article will be made available by the authors, without undue reservation.

## Ethics Statement

The studies involving human participants were reviewed and approved by Tokai University. The patients/participants provided their written informed consent to participate in this study.

## Author Contributions

DO, SY, TF, and YT contributed to conception/design of the study and organized the database. TF performed the statistical analysis. DO wrote the first draft of the manuscript. SY wrote sections of the manuscript. All authors contributed to manuscript revision, read, and approved the submitted version.

## Conflict of Interest

The authors declare that the research was conducted in the absence of any commercial or financial relationships that could be construed as a potential conflict of interest.

## Publisher's Note

All claims expressed in this article are solely those of the authors and do not necessarily represent those of their affiliated organizations, or those of the publisher, the editors and the reviewers. Any product that may be evaluated in this article, or claim that may be made by its manufacturer, is not guaranteed or endorsed by the publisher.

## Footnotes

^1^The local region and the club's CSR activities were not affected by the Covid-19 pandemic during the estimation period.

^2^Stage 6 involved reporting the results of the estimation and the research to MYFC. Although this phase plays an important role by showing the correctness and significance of the activities of MYFC, it is irrelevant to the results of the current study. Thus, it was omitted from this study.

^3^USD 1 = JPY 103.

^4^Adjusted to the target CSR activity (30-minute enjoyable soccer or physical activity program).

^5^1.5 h is the total amount of time to make a plan for the daily physical activity programs in their nursery school.

^6^Public servants' salary in each municipality (see Table 1) ×129 times ×2 staffs = USD 2,434.835.

^7^The deterioration of an outcome over time.

^8^The number of outcomes that would have occurred even if the program had not been conducted.

^9^The number of outcomes that has displaced other outcomes.
